# Sensitivity of cognitive function tests to acute hypoxia in healthy subjects: a systematic literature review

**DOI:** 10.3389/fphys.2023.1244279

**Published:** 2023-10-11

**Authors:** Titiaan E. Post, Laurens G. Heijn, Jens Jordan, Joop M. A. van Gerven

**Affiliations:** ^1^ German Aerospace Center (DLR), Institute of Aerospace Medicine, Cologne, Germany; ^2^ Centre for Human Drug Research (CHDR), Leiden, Netherlands; ^3^ Leiden Academic Centre for Drug Research, Leiden, Netherlands; ^4^ Medical Faculty, University of Cologne, Cologne, Germany; ^5^ Leiden University Medical Center, Leiden, Netherlands; ^6^ Central Committee on Research Involving Human Subjects (CCMO), The Hague, Netherlands

**Keywords:** acute normobaric hypoxia, acute hypobaric hypoxia, cognitive function, healthy volunteers, sensitivity

## Abstract

Acute exposure to hypoxia can lead to cognitive impairment. Therefore, hypoxia may become a safety concern for occupational or recreational settings at altitude. Cognitive tests are used as a tool to assess the degree to which hypoxia affects cognitive performance. However, so many different cognitive tests are used that comparing studies is challenging. This structured literature evaluation provides an overview of the different cognitive tests used to assess the effects of acute hypoxia on cognitive performance in healthy volunteers. Less frequently used similar cognitive tests were clustered and classified into domains. Subsequently, the different cognitive test clusters were compared for sensitivity to different levels of oxygen saturation. A total of 38 articles complied with the selection criteria, covering 86 different cognitive tests. The tests and clusters showed that the most consistent effects of acute hypoxia were found with the Stroop test (where 42% of studies demonstrated significant abnormalities). The most sensitive clusters were auditory/verbal memory: delayed recognition (83%); evoked potentials (60%); visual/spatial delayed recognition (50%); and sustained attention (47%). Attention tasks were not particularly sensitive to acute hypoxia (impairments in 0%–47% of studies). A significant hypoxia level-response relationship was found for the Stroop test (*p* = 0.001), as well as three clusters in the executive domain: inhibition (*p* = 0.034), reasoning/association (*p* = 0.019), and working memory (*p* = 0.024). This relationship shows a higher test sensitivity at more severe levels of hypoxia, predominantly below 80% saturation. No significant influence of barometric pressure could be identified in the limited number of studies where this was varied. This review suggests that complex and executive functions are particularly sensitive to hypoxia. Moreover, this literature evaluation provides the first step towards standardization of cognitive testing, which is crucial for a better understanding of the effects of acute hypoxia on cognition.

## Introduction

Hypoxemia is defined as low oxygen levels in the blood (Bhutta et al., 2022). It can occur in obstructive sleep apnea (OSA), in various pulmonary diseases such as COPD and COVID-19, in neuromuscular disorders like Guillain-Barré syndrome, Pompe’s disease or myasthenia gravis, and in central nervous system conditions like Alzheimer’s (Sivak et al., 1999; Orešič et al., 2011; Branson and Faarc, 2018; Chokesuwattanaskul et al., 2021; Rahman et al., 2021). Hypoxemia may also develop at high altitudes, where less oxygen is available due to the low atmospheric pressure (Mulroney et al., 2015).

Hypoxia research is divided into three arbitrary exposure designs: chronic, intermittent and acute hypoxia. Research on chronic hypoxia has shown the ability of the human body to adapt to prolonged exposure to low oxygen levels or pressures (Gilbert-Kawai et al., 2014). Hence, individuals residing at high altitudes or those who have had recent altitude exposure are frequently ineligible to participate in clinical trials investigating the impacts of hypoxia. In recent years, intermittent hypoxia (IH) has been studied, attempting to use the human body’s adaptability to IH as a therapeutic effect for various diseases (Navarrete-Opazo and Mitchell, 2014). This review will focus only on acute non-intermittent hypoxia.

Studying acute hypoxia is of particular importance in assessing the safety of occupational or recreational settings that involve acute exposure to high altitude, e.g., pilots, military personnel, mountaineers, etc. Hypoxia facilities can induce either hypobaric hypoxia by reducing barometric pressure or normobaric hypoxia by diluting oxygen fraction with nitrogen (DeHart and Davis, 2002). There has long been debate as to whether there is a difference in physiological response to isolated changes in barometric pressure, with normal levels of oxygen and carbon dioxide (Naughton et al., 2012). More recent studies suggest that there is indeed a significant impact of pressure alone (Savourey et al., 2003; Savourey et al., 2007; Millet et al., 2012; Coppel et al., 2015; McMorris et al., 2017).

One of the first symptoms of hypoxia exposure is impaired cognitive functioning. The level of oxygen saturation is considered to be the key predictor in determining the extend of cognitive impairment caused by hypoxia (McMorris et al., 2017). During high-altitude activities, cognitive performance plays a critical role in tasks that require attention, decision-making and memorization of protocols, as it can mitigate the risk of potential disasters. Over the years, numerous cognitive tests have been developed to evaluate cognitive performance. However, due to the wide range of cognitive tests used in studies examining cognitive performance during acute hypoxia exposure, it is challenging to compare results across different studies.

A useful test for the cognitive effects of acute hypoxia can be characterized as any response measure that shows a clear, consistent response to meaningful hypoxia levels, across studies from a sufficient number of different research groups. A hypoxia level-response relationship and a plausible relationship between the function test and the physiological response to hypoxia are indications that the test reflects physiological activity.

Previously, similar criteria were used to evaluate the usefulness of central nervous system (CNS)-tests for the effects of antipsychotic drugs (De Visser et al., 2001), benzodiazepines (De Visser et al., 2003), selective serotonin re-uptake inhibitors (SSRIs) (Dumont et al., 2005), 3,4-methyleendioxymethamfetamine (MDMA) (Dumont and Verkes, 2006), D9-tetrahydrocannabinol (THC) (Zuurman et al., 2009) and alcohol (Zoethout et al., 2011) in healthy subjects. In general, these systematic reviews showed that only a small number of tests actually display proper characteristics for a meaningful effect biomarker, and that these tests differ between drug classes (Van Gerven et al., 2019).

This review aims to provide an overview of the wide range of cognitive tests used to assess cognitive performance during acute hypoxia in healthy volunteers, to evaluate the differences in test sensitivity to different levels of hypoxia, to explore the role of barometric pressure, and to ultimately identify which tests best meets the criteria to serve as a meaningful test of the functional effects of hypoxia.

## Methods

### Structured literature evaluation

A literature search was performed up to 9 of November 2022 using Pubmed, Embase, Web of Science, Cochrane Library, Emcare, PsycINFO and Academic Search Premier. Key words used in the searches were combinations of “hypoxia”, “cognitive function”, “cognition”, “neuropsychological tests”, “biomarkers”, “cognitive dysfunction” and “mental deterioration”. The searches were limited to healthy adults and articles in English. The resulting studies were subject to several selection criteria. Reviews, studies in experimental animals or patients, ≤5 healthy subjects and confounding factors like exercise, acclimatization, sleep, brain stimulation and breathing exercise were excluded. The review was restricted to the effects of hypoxia exposure of <8 h and the presense of a normoxia condition, served as baseline or control group. Studies that contained a confounding factor but included both a normoxia and hypoxia group in the study design were eligible for analysis. Controlling the end-tidal CO_2_ can impact oxygen saturation, making isocapnia a potential confounding factor. Nevertheless, studies with isocapnic hypoxia were included when the oxygen saturation was documented.

### Mapping of results

Most studies compared hypoxia and normoxia groups, using one or more cognitive tests. In addition, some studies examined different exposure durations and/or severities. The normoxia condition represented either a placebo-controlled group or served as a baseline measurement of the group that would later be exposed to the hypoxia condition. Each primary test parameter for each cognitive test used in a study was considered a unique data point. Thus, a single study usually accounted for multiple data points. In some cases, there was no significant difference between groups for the primary effect parameter, while there was a significant difference for a secondary effect parameter. However, including these secondary effects would result in an overestimation of the cluster sensitivity. Hence, only primary effect parameters were considered.

All data points were collected in a Microsoft Excel^®^ database and recorded as a significant impairment or decrease (−), as no significant effect (=) or as a significant improvement or increase (+) of the test parameter during the hypoxia condition compared to the normoxia condition. The absolute values of the test effects or the levels of significant impairment were not included in the analysis. Consequently, this approach addresses the likelihood that a study with a hypoxia condition produces a statistically significant effect on a given cognitive test.

Study characteristics that were recorded in the database were: effect [+(improvement/increase)/= (no change)/- (deterioration/reduction)], test domain, test cluster, barometric pressure (hypobaric or normobaric), hypoxia intensity (“dose”, displayed as FiO_2_/altitude), dose normalization (four levels), oxygen saturation, exposure duration (including test duration), test duration, oxygen administration (mask/chamber/altitude), blinding (open/single-blind/double blind), randomization (randomized/non-randomized), control (baseline/control group), design (parallel/crossover), number of subjects, sex and age. Although blood CO_2_ levels are relevant for hypoxia research, they were not included in the database, as most studies did not document this information.

### Analysis of individual tests and test clusters

Cognitive tests with at least five data points (i.e., five different results from different experiments) were included in the analysis of the individual test results. Cognitive tests that were alternate versions of the original tests, or which tested the same cognitive functionality, were clustered to increase the number of evaluable data points. The clusters were divided into six domains for a better overview. The allocation of domains and clustering of cognitive tests was based on “A Compendium of Neurophysiological tests: Administration, Norms, and Commentary” (Strauss et al., 2006), adapted according to comparable systemic biomarker reviews that we performed previously for drug classes (De Visser et al., 2001; De Visser et al., 2003; Dumont et al., 2005; Dumont and Verkes, 2006; Zuurman et al., 2009; Zoethout et al., 2011). The sensitivity of a test was expressed as the percentage of statistically significant outcomes, relative to the total number of times that the test was used in the literature. The percentages of tests that showed cognitive impairment were calculated for each cluster.

### Level-response relationships

While analysis of individual tests and test clusters provides an overview of the sensitivity of different neuropsychological functions to hypoxia, this does not include information on the relationship between the level of hypoxia and the effect on cognitive test performance. This relationship was studied by first applying a four-level dose normalization to oxygen saturation. These four levels represent the severity of hypoxia as described by Castor and Borgvall (Castor and Borgvall, 2015) and based on Woodrow and Webb (Woodrow and Webb, 2011). The oxygen saturation levels are ≥90%, 89%–80%, 79%–70% and ≤69%. For studies that did not provide oxygen saturation, the fraction of inspired oxygen or altitude was used to estimate oxygen saturation (Woodrow and Webb, 2011; Castor and Borgvall, 2015). Only tests or clusters containing at least 10 data points were included in this analysis. Relationships were tested with simple linear regression analysis. Finally, a comparison was made between all similar tests/clusters that were measured both in normobaric hypoxia and hypobaric hypoxia. Similarly, these results were divided over the four levels of dose normalization.

## Results

### Study design

The literature search yielded 38 different studies on acute hypoxia that met all criteria, published between 1993 and 31 August 2022. The number of participants ranged from 6 to 50 and ages from 22 to 41 years (range of mean ages between different studies). In 40% of studies only healthy men were included, and 5% of studies included only women. Fifty-five percent of studies included men and women. A summary of the study characteristics is reported in Table 1.

**TABLE 1:** Summary of general study characteristics. M = male, F = female, M/F = male and female, NH = normobaric hypoxia, HH = hypobaric hypoxia, SpO_2_ = oxygen saturation, CAU = Caucasian individuals, AA = African-American.

Author	Participants (sex)	(Corresponding) altitude (NH/HH)	Exposure duration inclusive test time (test time)	SpO_2_% (SD, SEM, 95%CI)^a^	Design
Asmaro et al. (2013)	34 (M/F)	5334 m (HH)	30 min (5 min)	NA	baseline controlled
		7620 m (HH)	5 min (5 min)		
Beer et al. (2017)	12 (M/F)	5486 m (HH)	18 min (continuous)	NA	baseline controlled
	11 (M/F)	7620 m (HH)	5 min (continuous)		
Blacker and McHail (2021)	26 (M/F)	6096 m (NH)	10 min (10 min)	79.8% ± 6.9% (SD)	baseline
Blacker and McHail (2022)	30 (M/F)	6096 m (NH)	14.5 min (14 min)	∼75 ± 1.5% (SEM)	placebo crossover
Caldwell et al. (2018)	10 (M)	NA	25 min (NA)	79% ± 3% (SD)	placebo crossover
				88% ± 1% (SD)	
Chroboczek et al. (2022)	32 (M)	3800 m (NH)	35–36 min (5–6 min)	∼78 ± 4% (SD)	placebo crossover
Davranche et al. (2016)	11 (M)	4350 m (HH)	3–5 h (∼24 min)	83% ± 1.2% (SEM)	baseline controlled
Decroix et al. (2018)	20 (M/F)	4000 m (NH)	57 min (27 min)	83.8% ± 2.1% (SD)	placebo crossover
Falla et al. (2022)	48 (M/F)	3000 m (HH)	10 min (5 min)	93.6% ± 2% (SD)	placebo crossover
		5000 m (HH)		79.2% ± 4.8% (SD)	
Feeback et al. (2017)	6 (M) (CAU)	4400 m (NH)	<32 min (<2 min)	∼81 ± 2.3%	placebo crossover
			<62 min (<2 min)	79.5% ± 4.8% (SD)	
			<92 min (<2 min)	∼83 ± 9% (SD)	
			<117 min (<2 min)	∼83 ± 4.2% (SD)	
	6 (M) (AA)		<32 min (<2 min)	∼86 ± 4.7% (SD)	
			<62 min (<2 min)	∼86 ± 4.6% (SD)	
			<92 min (<2 min)	∼83 ± 4% (SD)	
			<117 min (<2 min)	∼85 ± 4.7% (SD)	
Gerhart et al. (2019)	10 (M)	3800 m (NH)	60 min (5 min)	∼85 ± 3%	placebo parallel
Hewett et al. (2010)	50 (M/F)	2438 m (NH)	45 min (30 min)	∼95 ± 1% (SEM)	placebo crossover
		3048 m (NH)		∼92 ± 1% (SEM)	
		3658 m (NH)		∼88 ± 1% (SEM)	
		4267 m (NH)		∼84 ± 1% (SEM)	
Kammerer et al. (2018)	11 (M/F)	3883 m (HH)	∼53 min (∼8 min)	84.8% ± 4.9% (SD)	baseline controlled
	7 (M/F)	3883 m% (NH)		82.9% ± 5.8% (SD)	
Kerr et al. (2022)	21 (M/F)	14% (NH)	60 min (5 min)	91% ± 3% (SD)	placebo crossover
			90 min (5 min)	90% ± 3% (SD)	
Kim et al. (2015)	8 (M)	4300 m (NH)	<32 min (<2 min)	87% ± 2% (SEM)	baseline controlled
Kourtidou-Papadeli et al. (2008)	10 (M/F)	2438 m (NH)	16 min (16 min)	92% ± 4.3% (SD)	baseline controlled
Lefferts et al. (2016)	20 (M)	4600 m (NH)	165 min (60 min)	75% ± 6% (SD)	baseline controlled
Legg et al. (2012)	25 (M)	2438 m (NH)	30 min (NA)	91% ± 2% (SD)	placebo crossover
			90 min (NA)		
Legg et al. (2016)	36 (M)	2438 m (HH)	39 min (12–15 min)	95% ± 3% (SD)%	placebo crossover
		3658 m (HH)		88% ± 3% (SD)	
Lei et al. (2019)	30 (F)	4000 m (NH)	12 min (2 min)	87% ± 6% (SD)	placebo crossover
Loprinzi et al. (2019)	21 (M/F)	4000 m (NH)	30 min (NA)	85% ± 1% (95%CI)	placebo crossover
Malle et al. (2016)	45 (M)	10000 m (NH)	3 min (3 min)	76% ± 0.8% (SEM)	placebo parallel
Nakata et al. (2017)	15 (M/F)	4400 m (NH)	40 min (5 min)	∼80 ± 10% (SD)	placebo crossover
Noble et al. (1993)	24 (M)	5850 m (NH)	30 min (25 min)	78% ± 2.9% (SD)	placebo parallel
Ochi et al. (2018)	14 (M/F)	3500 m (NH)	16.5 min (6.5 min)	86.2% ± 1.2% (SEM)	placebo crossover
Ogoh et al. (2018)	14 (M/F)	4400 m (NH)	45 min (5 min)	80% ± 10% (SD)	baseline controlled
Reményi et al. (2018)	33 (M/F)	4000 m (HH)	20 min (15 min)	∼86 ± 4.5% (SD)%	baseline controlled
Rossetti et al. (2021)	24 (M/F)	4500 m (NH)	210 min (90 min)	82% ± 2% (95%CI)	placebo crossover
Seech et al. (2020)	39 (M/F)	5400 m (NH)	1–9 min (continuous)	∼81 ± 0.8% (SEM)	placebo crossover
			10–18 min (continuous)	∼74 ± 1% (SEM)	
			19–27 min (continuous)	∼73 ± 1% (SEM)	
Seo et al. (2015)	16 (M)	4300 (NH)	60 min (5 min)	∼82 ± 2.5%	baseline
Seo et al. (2017)	15 (F)	4300 m (NH)	30 min (NA)	83.3% ± 5.1% (SD)	baseline controlled
			60 min (NA)	82.2% ± 5.1% (SD)	
Stepanek et al. (2013)	25 (M/F)	7101 m (NH)	<5 min (<2 min)	75.8% ± 8.3% (SD)	baseline controlled
Turner et al. (2015b)	15 (M/F)	5850 m (NH)	90 min (75 min)	80% ± 10% (SD)	placebo crossover
Turner et al. (2015a)	22 (M/F)	5850 m (NH)	90 min (40 min)	75% ± 1% (SEM)	placebo parallel
Valk et al. (2016)	24 (M)	2438 m (HH)	1–6 h (20 min)	93% (range 85%–95%)	baseline controlled
van der Post et al. (2002)	12 (M/F)	NA	130 min (100 min)	80.3% ± 1.2% (SD)	placebo crossover
				90% ± 0.9% (SD)	
Wang et al. (2013)	10 (M)	3560 m (HH)	6.5 h (30 min)	NA	baseline controlled
Williams et al. (2019)	12(M)	4500 m (NH)	60 min (<5 min)	∼81 ± 4% (SD)	placebo crossover
		3000 m (NH)		∼90 ± 1.5% (SD)	
		1600 m (NH)		∼94 ± 1.3 (SD)%	

^a^ SpO_2_ levels with the '∼' symbol, were estimated from a graphical representation.

Forty-seven percent of the reviewed studies had an open design; 37% were single-blinded; 5% were double blinded and for 11% the blinding was unknown. In addition, a majority of the studies had a crossover design (89%) and 11% had a parallel design. Normoxia served in 61% of studies as a control group and in 39% as a baseline measurement.

### Atmospheric conditioning of hypoxia

Eighteen studies were performed in a hypoxic chamber (Asmaro et al., 2013; Kim et al., 2015; Seo et al., 2015; Lefferts et al., 2016; Legg et al., 2016; Valk et al., 2016; Beer et al., 2017; Feeback et al., 2017; Seo et al., 2017; Decroix et al., 2018; Reményi et al., 2018; Gerhart et al., 2019; Williams et al., 2019; Seech et al., 2020; Blacker and McHail, 2021; Blacker and McHail, 2022; Chroboczek et al., 2022; Falla et al., 2022; Kerr et al., 2022), 16 with a breathing mask that induced hypoxia (Turner et al., 2015a; Noble et al., 1993; Ochi et al., 2018; Malle et al., 2016; Lei et al., 2019; Caldwell et al., 2018; Hewett et al., 2010; Legg et al., 2012; Ogoh et al., 2018; Stepanek et al., 2013; Nakata et al., 2017; Loprinzi et al., 2019; Kourtidou-Papadeli et al., 2008; Turner et al., 2015b), two studies were performed at altitude (Wang et al., 2013; Davranche et al., 2016), one both at altitude and in a chamber (Kammerer et al., 2018), and one both in a chamber and with a breathing mask (Rossetti et al., 2021). The exposure duration ranged from 10 min to 6.5 h with 92% of studies using durations of <3 h. The data points obtained were divided into exposure to normobaric hypoxia (133; 67%) and hypobaric hypoxia (66; 33%). The mean altitude and mean oxygen saturation for normobaric and hypobaric hypoxia were 4500 m and 84.4%, and 3300 m and 86.9%, respectively.Only two s tudies were performed with isocapnic hypoxia, so this condition was not analysed separately (Caldwell et al., 2018).

### Tests, clusters and domains

A total of 86 different tests were used. Only six tests (including comparable variants) (7%) were used five times or more and consequently produced five or more data points for the analysis of hypoxia on individual tests. These most frequently used tests were the Trail making test A (14), Trail making test B (14), Stroop test (12), Finger tapping (5), Go/no Go (5) and Digit symbol substitution test (DSST) (5), shown in Table 2. Overall, the Stroop test and Finger tapping test showed the highest sensitivity to acute hypoxia (which produced significant effects in 42% and 40% of experiments, resp.), followed by Trail making B (29%), Trail making A (21%) and the Go/no Go task (20%). The DSST did not show any significant cognitive impairment.

**TABLE 2:** A summary of the most frequently used tests (≥5 times) for measuring the effects of acute hypoxia on cognitive performance. The table shows the number of times tests were, and in brackets, the number of studies from which these data points were collected. The test performance is indicated by significant impairment or decrease (−), no significant effect (=) or significant improvement or increase (+). Test sensitivity was calculated as the number of times a test showed a significant impairment out of the total number of times the test was taken.

Test name	Number of times taken (number of studies)	Test performance hypoxia vs. normoxia			Test sensitivity (%)
		**-**	=	+	
Stroop	12 (9)	5	7	0	42
Finger tapping	5 (4)	2	3	0	40
Trail making B	14 (5)	4	10	0	29
Trail making A	14 (5)	3	11	0	21
Go/no Go	5 (5)	1	4	0	20
DSST	5 (4)	0	5	0	0

A cluster analysis was performed because of the small number of tests that were performed frequently enough to allow an individual test analysis. Tests used only incidentally were clustered together with other tests that require the same cognitive functionality. This allowed us to increase the sample size and thus perform a more reliable analysis on how hypoxia affects different cognitive functions. The tests were grouped into 32 functional clusters, covering six main neurocognitive domains (Table 3). Fourteen of the 32 clusters contained at least five data points and were used for further analysis of overall hypoxia effects (Table 4). Both delayed recognition clusters auditory/verbal memory (83%) and visual/spatial memory (50%), and the evoked potential cluster (60%) had the highest sensitivity to acute hypoxia (irrespective of level). In contrast, immediate recognition tests showed virtually no significant effects (auditory/verbal memory tests (20%); visual/spatial memory tests (0%)). In addition to delayed recognition and evoked potential, the clusters of sustained attention (47%), motor control (40%) and divided attention (36%) showed a higher sensitivity for hypoxia. The clusters inhibition (26%), shifting (23%) and reaction time (19%) rarely yielded significant results, whereas in the literature, tests from these clusters were used most frequently (inhibition 23/182, shifting 31/182, and reaction time 21/182 tests).

**TABLE 3:** An overview of the 86 tests condensed into domains and clusters.

Domain	Cluster	Test
Attention	Divided Attention	Auditory monitoring task, Combined distributive attention test, Divided attention test, Recourse management task, System monitoring, Visual monitoring task
	DSST-like	Digit symbol substitution test
	Focused/selective attention	Shifting attention test
	Reaction time	Choice reaction time, Deary–Liewald reaction time task, Go/No-go task, Simple auditory and visual reaction times, Simple unprepared reaction time, Sorted reaction test, Target reaction test, The binary choice task
	Sustained attention	Behavioural tracking task, Continuous performance test, Motion detection task, Paced auditory serial addition task 1.2s, Paced auditory serial addition task 1.6s, Psychomotor vigilance task, Tracking task, Vigilance and tracking task
Executive	Inhibition	Eriksen flanker test, Go/No-go task, Simon task, Stroop test, Verbal interference, Visual interference
	Language	King-Devick test
	Planning	Tower puzzle
	Reasoning/association	Abstract matching, Complex logical reasoning task, Logical relations, Math task, Mathematical processing, Pathfinder combined, Symbol digit coding, SYNWIN math task
	Reward	Balloon analogue risk test
	Shifting	Switching of attention, The Wisconsin card sorting task, Trail making test A, Trail making test B
	Spatial orientation	Letter rotation test, Line orientation test, Manikin test
	Working memory	3-back, Corsi block-tapping task, Digit span backward test, Digit span forward test, Maze, N-back, Operation span task, Visual sequence comparison
Memory	Auditory/verbal memory: delayed recall	Free recall test, Memory interference task, Multiple memory task
	Auditory/verbal memory: delayed recognition	Emotion recognition task, Memory recognition task, Verbal memory test, Serial recognition of words
	Auditory/verbal memory: immediate recognition	Memory search, Running memory continuous performance task
	Visual/spatial memory: delayed recall	Digital tachistoscopy
	Visual/spatial memory: delayed recognition	Memory task, Serial recognition of figures, Sternberg short-term memory task, Visual memory task, Visual object learning test
	Visual/spatial memory: immediate recognition	Matching to sample, Visual searching task
Motor	Motor control	Finger tapping test
	Visuo-motor control	Motor praxis test, Gridshot
Neurophysiological	Evoked potential	Auditory N100, Auditory P200, Auditory P50, Electroencephalogram, N140, P300, Visual P100
Subjective experience	Scale anger	Mood test
	Scale anxiety	Mood test
	Scale depression	Mood test
	Scale fatigue	Mood test
	Scale happiness	Mood test
	Scale restlessness	Mood test
	Scale sleepiness	Mood test, Stanford sleepiness scale
	Scale vigilance	Mood test, Level of vigilance visual analogue scale
	Scale vigour	Mood test
	Total mood	Profile of mood state, Mood test

**TABLE 4:** An overview of clusters in which at least five times a test was taken. The table shows the number of times tests were taken in each cluster and, in brackets, the number of studies from which these tests were collected. The test performance is indicated by significant impairment or decrease (−), no significant effect (=) or significant improvement or increase (+). Cluster sensitivity was calculated as the number of times a test showed a significant impairment out of the total number of times the test was taken.

Domain	Cluster	Number of times taken (number of studies)	Test performance hypoxia vs. normoxia			Cluster sensitivity
			**-**	=	+	
Attention	Sustained attention	17 (12)	8	9	0	47%
	Divided attention	11 (5)	4	7	0	36%
	Reaction time	21 (9)	4	16	1	19%
	DSST-like	5 (4)	0	5	0	0%
Executive	Reasoning/association	17 (8)	6	11	0	35%
	Working memory	17 (7)	5	12	0	29%
	Inhibition	23 (16)	6	16	1	26%
	Shifting	31 (7)	7	24	0	23%
Memory	Auditory/verbal memory: delayed recognition	6 (4)	5	1	0	83%
	Visual/spatial memory: delayed recognition	8 (6)	4	4	0	50%
	Auditory/verbal memory: immediate recognition	5 (3)	1	4	0	20%
	Visual/spatial memory: immediate recognition	6 (2)	0	6	0	0%
Motor	Motor control	5 (4)	2	3	0	40%
Neurophysiological	Evoked potential	10 (4)	6	4	0	60%

### Level-response relationship

**FIGURE 1:**
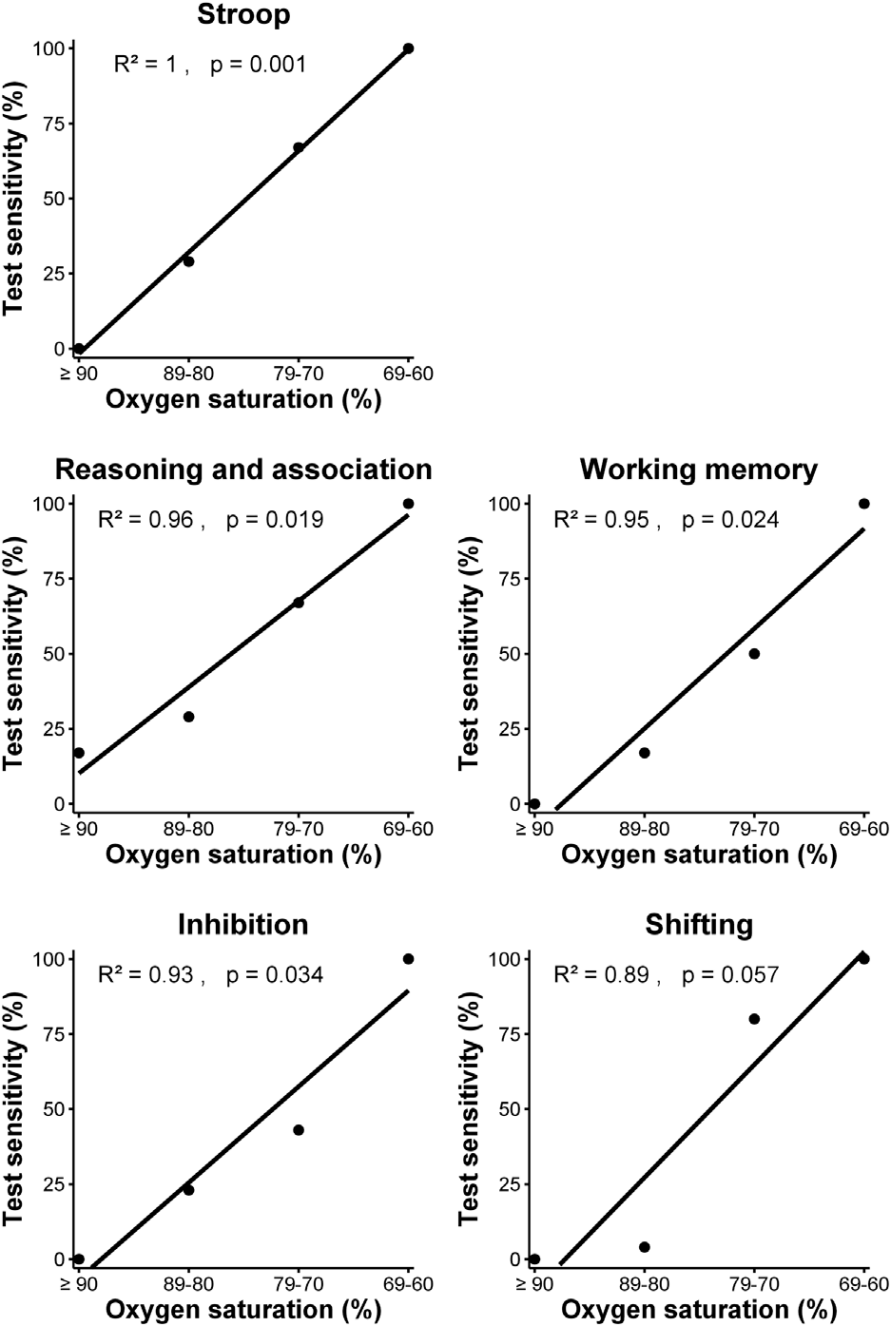
Relationship between test sensitivity and oxygen saturation of Stroop test and clusters within the executive domain using simple linear regression analysis.

Individual tests and clusters with ≥10 datapoints were inspected for potential level-response relationships (Table 5; Table 6). (Near) significant associations were found for the Stroop test, Trail making A, Trail making B and 25% of clusters. The Stroop test (*p* = 0.001, *R* ^2^ = 1.00) and clusters within the executive domain showed a significant relationship (reasoning/association *p* = 0.019, *R* ^2^ = 0.96; working memory *p* = 0.024, *R* ^2^ = 0.95; and inhibition *p* = 0.035, *R* ^2^ = 0.93) or trend (shifting *p* = 0.057, *R* ^2^ = 0.89) between the four defined levels of oxygen saturation and test sensitivity, with lower saturations more often leading to a higher test sensitivity (Figure 1). The Trail making A, Trail making B and clusters in the attention and neurophysiological domain did not show a level-response relationship between oxygen saturation and test sensitivity.

**TABLE 5:** The level-response relationship between oxygen saturation and sensitivity for tests used ≥10 times. Oxygen saturation is normalized into four groups: ≥90%, 89%–80%, 79%–70% and 69%–60%. The table shows the number of times tests were taken and, in brackets, the number of studies from which these tests were collected. The test performance is indicated by significant impairment or decrease (−), no significant effect (=) or significant improvement or increase (+). Test sensitivity was calculated as the number of times a test showed a significant impairment out of the total number of times the test was taken.

Test name	SpO_2_ normalization	Tests (studies)	Test performance hypoxia vs. normoxia			Test sensitivity
			**-**	=	+	
Stroop	Total	12 (9)	5	7	0	42%
	≥90%	1 (1)	0	1	0	0%
	89%–80%	7 (6)	2	5	0	29%
	79%–70%	3 (3)	2	1	0	67%
	69%–60%	1 (1)	1	0	0	100%
Trail making B	Total	14 (5)	4	10	0	29%
	≥90%	0 (0)	0	0	0	0%
	89%–80%	11 (3)	0	11	0	0%
	79%–70%	2 (2)	2	0	0	100%
	69%–60%	1 (1)	1	0	0	100%
Trail making A	Total	14 (5)	3	11	0	21%
	≥90%	0 (0)	0	0	0	0%
	89%–80%	11 (3)	1	10	0	9%
	79%–70%	2 (2)	2	0	0	100%
	69%–60%	1 (1)	1	0	0	100%

**TABLE 6:** The level-response relationship between oxygen saturation and sensitivity of clusters tested ≥10 times. Oxygen saturation is normalized into four groups: ≥90%, 89%–80%, 79%–70% and 69%–60%. The table shows the number of times tests were taken in each cluster and, in brackets, the number of studies from which these tests were collected. The test performance is indicated by significant impairment or decrease (−), no significant effect (=) or significant improvement or increase (+). Cluster sensitivity was calculated as the number of times a test showed a significant impairment out of the total number of times the test was taken.

Domain	Cluster	SpO_2_ normalization	Tests (studies)^a^	Test performance hypoxia vs. normoxia			Cluster sensitivity
				**-**	=	+	
Attention	Sustained attention	Total	17 (12)	8	9	0	47%
		≥90%	2 (2)	0	2	0	0%
		89%–80%	10 (7)	6	4	0	60%
		79%–70%	5 (5)	2	3	0	40%
	Divided attention	Total	11 (5)	4	7	0	36%
		≥90%	4 (3)	1	3	0	25%
		89%–80%	3 (2)	0	3	0	0%
		79%–70%	2 (1)	1	1	0	50%
		69%–60%	2 (1)	2	0	0	100%
	Reaction time	Total	21 (9)	4	16	1	19%
		≥90%	6 (2)	1	5	0	17%
		89%–80%	12 (7)	2	9	1	17%
		79%–70%	3 (2)	1	2	0	33%
Executive	Reasoning/association	Total	17 (8)	6	11	0	35%
		≥90%	6 (3)	1	5	0	17%
		89%–80%	7 (5)	2	5	0	29%
		79%–70%	3 (3)	2	1	0	67%
		69%–60%	1 (1)	1	0	0	100%
	Working memory	Total	17 (7)	5	12	0	29%
		≥90%	5 (3)	0	5	0	0%
		89%–80%	6 (5)	1	5	0	17%
		79%–70%	4 (2)	2	2	0	50%
		69%–60%	2 (1)	2	0	0	100%
	Inhibition	Total	23 (16)	6	16	1	26%
		≥90%	3 (2)	0	3	0	0%
		89%–80%	13 (12)	3	10	0	23%
		79%–70%	7 (5)	3	3	1	43%
		69%–60%	1 (1)	1	0	0	100%
	Shifting	Total	31 (7)	7	24	0	23%
		≥90%	1 (1)	0	1	0	0%
		89%–80%	23 (4)	1	22	0	4%
		79%–70%	5 (3)	4	1	0	80%
		69%–60%	2 (1)	2	0	0	100%
Neurophysiological	Evoked potential	Total	10 (4)	6	4	0	60%
		89%–80%	6 (3)	5	1	0	83%
		79%–70%	4 (1)	1	3	0	25%

^a^ Some studies included multiple SpO_2_ levels in their experimental design.

Since sustained- and divided attention were sensitive to hypoxia, while also playing a role in executive functioning and memory, the contribution of attention deficits to reduced cognitive functioning was explored in more detail. At an oxygen saturation level of ≥90%, tests measuring sustained- and divided attention demonstrated sensitivity ranging from 0% to 25%, respectively, while the executive domain showed sensitivity between 0% and 17%. At an oxygen saturation level of 80%–90%, sustained attention showed a test sensitivity of 60%, whereas divided attention did not show any sensitivity, in contrast to the executive domain where sensitivity ranged from 4% to 29%. At an oxygen saturation level of 70%–80%, the sensitivity of sustained attention was 40%, while divided attention showed a 50% sensitivity, compared to the executive domain which ranged from 43% to 80%. Although sustained attention was not measured between an oxygen saturation level of 69%–60%, divided attention and all clusters within the executive domain demonstrated 100% sensitivity. There appears to be a cautious relationship between hypoxia-related declines in divided attention and executive performance (*p* = 0.059).

### The effect of barometric pressure

Table 7 presents the effects of normobaric and hypobaric hypoxia on overall cognitive performance for the four levels of oxygen saturation. At oxygen saturation above 80%, almost no differences in sensitivity were observed between normobaric and hypobaric hypoxia (4% and 5% at SpO_2_ ≥ 90% and 27% and 27% at SpO_2_ 89%–80%, respectively). At an oxygen saturation between 79% and 70%, hypobaric hypoxia affected cognitive performance more often (67%) than normobaric hypoxia (55%), although not significantly. At an oxygen saturation between 69% and 60%, no data were reported for normobaric hypoxia.

**TABLE 7:** Effects of barometric pressure on test sensitivity in all clusters. The table shows the number of times tests were taken and, in brackets, the number of studies from whish these tests were collected. Oxygen saturation is normalized into four groups: ≥90%, 89%–80%, 79%–70%, and 69%–60%. The number of times a significant effect was found is indicated by impairment or decrease (−), no significant effect (=) or improvement or increase (+).

Barometric pressure	SpO_2_ normalization	Tests (studies)^a^	Test performance hypoxia vs. normoxia			Test sensitivity
			**-**	=	+	
Normobaric	Total	133 (28)	39	92	2	29%
	≥90%	23 (5)	1	22	0	4%
	89%–80%	79 (18)	21	57	1	27%
	79%–70%	31 (7)	17	13	1	55%
Hypobaric	Total	66 (8)	25	41	0	38%
	≥90%	19 (3)	1	18	0	5%
	89%–80%	26 (5)	7	19	0	27%
	79%–70%	12 (3)	8	4	0	67%
	69%–60%	9 (2)	9	0	0	100%

^a^ Some studies included multiple SpO_2_ levels in their experimental design.

Given that a level-response relationship between oxygen saturation and test sensitivity occurred solely in clusters belonging to the executive domain, the comparison between normobaric hypoxia and hypobaric hypoxia was narrowed to this particular domain (Table 8). Oxygen saturation levels of ≥90% and between 89% and 80% did not yield significantly different effects on cognitive performance between normobaric hypoxia and hypobaric hypoxia. The largest difference was found at an oxygen saturation between 79% and 70% (50% and 86%, respectively), but this difference was not statistically significant.

**TABLE 8:** Effects of normobaric and hypobaric hypoxia on test sensitivity in the executive domain. The table shows the number of times tests were taken and, in brackets, the number of studies from whish these tests were collected. Oxygen saturation is normalized into four groups: ≥90%, 89%–80%, 79%–70% and 69%–60%. The number of times a significant effect was found is indicated by impairment or decrease (−), no significant effect (=) or improvement or increase (+).

Barometric pressure	SpO_2_ normalization	Tests (studies)^a^	Test performance hypoxia vs. normoxia			Test sensitivity
			**-**	=	+	
Normobaric	Total	62 (19)	11	50	1	18%
	≥90%	10 (3)	0	10	0	0%
	89%–80%	40 (14)	5	35	0	13%
	79%–70%	12 (4)	6	5	1	50%
Hypobaric	Total	28 (7)	14	14	0	50%
	≥90%	6 (2)	1	5	0	17%
	89%–80%	9 (4)	1	8	0	11%
	79%–70%	7 (3)	6	1	0	86%
	69%–60%	6 (2)	6	0	0	100%

^a^ Some studies included multiple SpO_2_ levels in their experimental design.

## Discussion

A large number of tests were used in the literature to measure the acute CNS effects of hypoxia in healthy adults. As with similar reviews for drug classes (De Visser et al., 2001; De Visser et al., 2003; Dumont et al., 2005; Dumont and Verkes, 2006; Zuurman et al., 2009; Zoethout et al., 2011), there were more tests than studies: 86 in 38 studies. Only six individual tests, including their variants, (7%) were used five times or more and consequently provided sufficient data points for our individual test analysis. This wide variety of tests limits cross-study comparison. This limitation not only hampers the current review, but a virtually unrestricted use of a large number of distinct neurocognitive tests also thwarts the field of hypoxia research as a whole. Although it may be useful to study different distinct areas of cognitive performance, interpretations and insights would strongly benefit from standardization within cognitive domains and test clusters. Moreover, standardization of exposure protocols would also improve comparisons between studies. Given this suboptimal context, we grouped tests into test clusters and functional domains, to obtain more insights into potentially meaningful hypoxia CNS-effects and protocols. Prior reviews demonstrated that this approach is useful for assessing function tests or biomarkers for drug effects (De Visser et al., 2001; De Visser et al., 2003; Dumont et al., 2005; Dumont and Verkes, 2006; Zuurman et al., 2009; Zoethout et al., 2011). While this methodology inevitably results in the loss of some information, it ultimately yields a structured and comprehensive overview of the chance of measuring significant CNS effects of acute hypoxia in studies with healthy adults.

Attention is an important functional domain that underlies most of the other CNS-performance tasks. Hypoxia has an effect on attention, particularly, at levels below 80%, which caused significant abnormalities of divided or sustained attention in 36% and 47% of studies, respectively. This effect of hypoxia on more demanding (particularly, divided) attention tests may also have influenced other complex or multifunctional tasks, particularly those in the executive domain. These clusters represent aspects of cognition with a direct effect on safety for military personnel and pilots. Especially, impairments in sustained attention, which were already frequently observed at an oxygen saturation between 89% and 80% (60%), could lead to serious safety risks when military personnel have to stand guard at altitude (2438 m–4572 m) on hostile territory. Therefore, this aspect of cognition should be carefully considered during training and preparation for a mission or flight. The effect of hypoxia on reaction time seems limited, even under more severe hypoxic conditions (SpO_2_ < 80%) a low-test sensitivity is observed (33%). However, it should be noted that in some cognitive tests, secondary effects on reaction time were observed, which were not included in the analysis to prevent bias.

In the executive domain, the different test clusters rarely showed an effect of mild hypoxia (SpO_2_ ≥ 80%). However, when oxygen saturation fell below 80%, the test sensitivity increased. The level-response relationship between oxygen saturation and test sensitivity could indicate that cognitive testing of the executive domain gives the most accurate representation of the physiological response of hypoxia. Although the mechanism causing the impairment of executive function by hypoxia is not clearly understood, this could be related to impaired concentration or (divided) attention, which are required during performance of any executive task. It is also possible that hypoxia-induced executive impairment is related more directly to decreased neural activity of the prefrontal cortex (Bishop et al., 2010; Shaked et al., 2018; Rosen et al., 2019). A review by Beebe and Gozal linked executive impairment of OSA patients to prefrontal cortex dysfunction (Beebe and Gozal, 2002). To our knowledge, however, only one study has examined the role of the prefrontal cortex in reducing executive function after hypoxia exposure in healthy individuals (Ochi et al., 2018). Although this study by Ochi et al. found a statistically significant relationship between impaired Stroop test performance and lower dorsolateral prefrontal cortex activation after a combined intervention of exercise and hypoxia, this relationship was not significant in hypoxia at rest. However, the oxygen saturation was also lower in the combined exercise and hypoxia intervention than in the hypoxia at rest intervention, which is expected as, exercise acutely decreases the oxygen saturation both combined with and independent of hypoxia (Woorons and Richalet, 2021). Therefore, a similar effect on the lower dorsolateral prefrontal cortex might be found for more severe hypoxia. Further research into the interaction between hypoxia, prefrontal cortex activation and executive function could help to get a better understanding of the physiological response to hypoxia.

Within the inhibition cluster, the Stroop test more often showed impairments (42%) than when other inhibition tests were used (mean of 26%). This may reflect the dependence of the performance of the Stroop test on accurate colour discrimination. Although this systematic review did not specifically search for visual function studies, the retina has been reported to be highly sensitive to hypoxia (Selvam et al., 2018). Based on this observation we recommend choosing the Stroop test over other inhibition tasks.

In the memory domain, delayed recognition was the most sensitive to hypoxia. Delayed auditory/verbal recognition tests showed the highest sensitivity among all clusters (83%). Delayed recognition is an aspect that can be highly relevant while navigating through new terrain. Therefore, this should be considered especially for exploratory expeditions at altitude.

Although the number of studies using neurophysiological tests during acute hypoxia was limited, our data suggest that tests within the neurophysiological domain could be a particularly sensitive tool to assess CNS functions during hypoxia. At an oxygen saturation between 89% and 80%, the evoked potential cluster showed higher sensitivity than any other cluster. This is in line with previous research by Tsarouchas et al. which concluded that evoked brain responses can be used for early detection of cognitive alterations during exposure to moderate hypobaric hypoxia (Tsarouchas et al., 2008).

The impact of barometric pressure on the severity of hypoxia is a subject of debate (Savourey et al., 2003; Savourey et al., 2007; Millet et al., 2012; Naughton et al., 2012; Coppel et al., 2015; McMorris et al., 2017). In light of this, a secondary objective of this review was to explore the effects of barometric pressure on cognitive function. No significant difference was found in test sensitivity due to barometric pressure in the overall analysis at all oxygen saturation levels. Similarly, when only the (complex) attention or executive function domains were considered, no significant difference was found. Although the number of studies was too small to be conclusive, this review does not provide indications that barometric pressure has a large impact on hypoxia sensitivity. This supports the traditional consensus that normobaric and hypobaric hypoxia can be used interchangeably (Naughton et al., 2012). However, more recent findings of several studies indicate that normobaric and hypobaric hypoxia have different physiological effects (Savourey et al., 2003; Savourey et al., 2007; Millet et al., 2012; Coppel et al., 2015; McMorris et al., 2017). Coppel et al. suggested that the traditional view might be based on the barometric pressure only showing effects for longer exposure times (>3 h) (Coppel et al., 2015). This could explain why this review of acute hypoxia showed no effect of barometric pressure, as only 8% of our studies used exposure times of more than 3 h, and only one of these tested the executive domain.

One limitation of this study was its reliance on characterizing the literature based on inspired oxygen fraction or altitude levels. This approach is probably hiding relatively large interindividual SpO_2_ variability and therefore mixing within single studies participants with very different hypoxemic levels. This limitation is inherent to experiments where oxygen saturations are not individually controlled, which constituted the vast majority of the studies in this review. To address this limitation and provide a clearer understanding of the results, we expressed the variability in Table 1 within each study by providing measures such as standard deviation (SD), standard error of the mean (SEM), or 95% confidence interval (95% CI).

An associated limitation of this review may be the variation in the distribution and heterogeneity of SpO_2_ levels between different tests or clusters, which were derived from a heterogeneity of different studies. This disparity in SpO_2_ levels may account for the observed differences in sensitivity to hypoxia among these clusters. For instance, clusters with studies encompassing a wide range of hypoxic levels may have an increased likelihood of demonstrating higher sensitivity to hypoxia severity, whereas clusters with studies investigating a narrow range of hypoxic levels may have reduced chances of showing hypoxia sensitivity. Consequently, this effect could introduce bias in the analysis of the hypoxic-dose response.

A third limitation of this study is that the majority of included studies were not blinded. This lack of blinding may introduce bias, as participants and researchers being aware of the hypoxic or normoxic exposure could inadvertently influence cognitive performance assessments.

All these factors reflect variabilities and disparities between hypoxia studies, which limits the conclusions that can be reached from the literature, and the selection of the most sensitive and reliable tests of cognitive effects of acute hypoxia. This emphasizes the need for more standardisation of methodologies, to improve the comparability and generalizability of hypoxia experiments.

## Conclusion

A large variety of cognitive tests were used in the literature to assess the effects of acute hypoxia on cognition in healthy adults. This huge methodological diversity is a major detriment to the investigation of the CNS effects of hypoxia. With these limitations, some suggestions can be made. The Stroop test as well as the clusters of sustained attention, auditory/verbal: delayed recognition, visual/spatial: delayed recognition, and evoked potential showed higher sensitivity than other tests and clusters. All clusters within the executive domain with more than 10 data points showed a clear level-response relationship or trend, with more frequent impairments at more severe levels of hypoxia. The data in our review showed no different physiological effect between hypobaric hypoxia and normobaric hypoxia. To further improve our understanding of the effects of acute hypoxia on cognition, standardization of exposure protocols and cognitive testing is crucial.

## Data Availability

The original contributions presented in the study are included in the article, further inquiries can be directed to the corresponding author.
